# Lipid Droplet Surface Promotes 3D Morphological Evolution of Non‐Rhomboidal Cholesterol Crystals

**DOI:** 10.1002/advs.202409201

**Published:** 2024-11-08

**Authors:** Hyun‐Ro Lee, Seunghan Kang, Siyoung Q. Choi

**Affiliations:** ^1^ Department of Chemical and Biomolecular Engineering Korea Advanced Institute of Science and Technology (KAIST) Daejeon 34141 Republic of Korea; ^2^ Advanced Battery Center KAIST Institute for the NanoCentury Korea Advanced Institute of Science and Technology (KAIST) Daejeon 34141 Republic of Korea

**Keywords:** cholesterol, crystal morphology, crystallization, interfacial phenomena, lipid droplets

## Abstract

Cholesterol crystals, which cause inflammation and various diseases, predominantly grow in a platy, rhomboid structure on the plasma membranes but exhibit an uneven three‐dimensional (3D) architecture intracellularly. Here, it is demonstrated how cholesterol crystallizes in a non‐rhomboidal shape on the surface of lipid droplets and develops into 3D sheet‐like agglomerates using an in vitro lipid droplet reconstitution system with stereoscopic fluorescence imaging. The findings reveal that interfacial cholesterol transport on the lipid droplet surface and unique lipid droplet components significantly influence the nucleation‐and‐growth dynamics of cholesterol crystals, leading to crystal growth in various polygonal shapes. Furthermore, cholesterol crystals readily agglomerate to form large, curved sheet structures on the confined, spherical surfaces of lipid droplets. This discovery enhances the understanding of the volumetric morphological growth of intracellular cholesterol crystals.

## Graphical abstract

This study reveals how cholesterol crystallizes uniquely on lipid droplet surfaces, forming non‐rhomboidal, platy crystals and three‐dimensional (3D) sheet‐like crystal agglomerates. It highlights how interfacial cholesterol transport, lipid composition, and droplet geometry influence the cholesterol crystallization dynamics and crystal shape. This provides key insights into the diverse morphologies of intracellular cholesterol crystals that induce inflammation and mechanical cell damage.

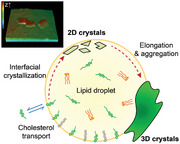

## 1 Introduction

Cholesterol is essential for modulating cell signaling, maintaining cellular membrane integrity, and producing steroid hormones, vitamin D, and bile acids.^[^ ^1^ ^]^ However, excessive cholesterol generates water‐insoluble, solid crystals that trigger mechanical tissue damage and inflammatory responses, potentially causing fatal clinical disorders such as atherosclerotic cardiovascular diseases,^[^ ^2^, ^3^ ^]^ demyelinating disorders,^[^ ^4^, ^5^ ^]^ fibrosing nonalcoholic steatohepatitis,^[^ ^6^ ^]^ and age‐related macular degeneration.^[^ ^7^ ^]^ Because of its close relationship with wide‐ranging diseases, the underlying mechanisms by which cholesterol crystallizes in human bodies have been extensively studied to understand pathogenesis and identify new treatments. It has been traditionally proposed that cholesterol crystals nucleate on the lipid bilayer membranes, such as plasma membranes, lysosomes, and multilamellar bodies, at high cholesterol levels through macrophage models^[^ ^3^, ^8^, ^9^, ^10^ ^]^ and synthetic lipid bilayers.^[^ ^11^, ^12^, ^13^, ^14^ ^]^ When cholesterol levels exceed the solubility limit in the bilayer membranes, cholesterol forms crystalline cholesterol nanodomains and further grows into triclinic or monoclinic cholesterol monohydrate crystals.^[^ ^15^, ^16^ ^]^ A triclinic polymorph is thermodynamically stable and has a rhomboidal platy shape, while a monoclinic one is metastable and grows in different shapes, such as helixes, tubes, rods, or plates.^[^ ^8^, ^11^, ^12^, ^13^ ^]^ Cholesterol crystals can grow as triclinic or monoclinic polymorphs depending on the kinetic effects,^[^ ^17^ ^]^ tissue types,^[^ ^18^ ^]^ nucleation sites,^[^ ^8^ ^]^ phospholipids,^[^ ^12^, ^13^, ^19^ ^]^ and hydration levels,^[^ ^20^ ^]^ but rhomboid‐like triclinic crystals predominate over time as the monoclinic form is transformed into the more stable triclinic one.

**Figure 1:**
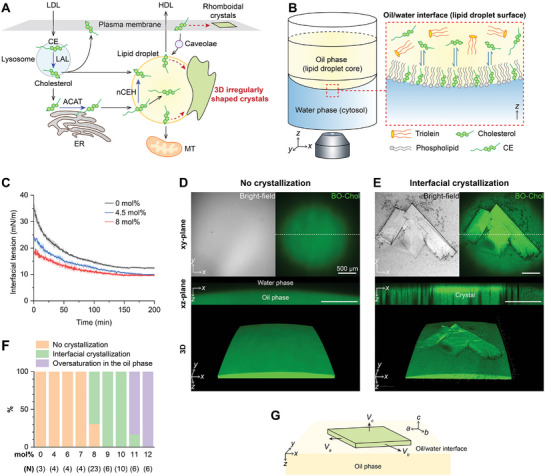
Cholesterol crystallization on the model lipid droplet surface. A) Intracellular cholesterol processing. Cholesteryl esters (CEs) enter the cell via low‐density lipoproteins (LDLs) and are hydrolyzed into free cholesterol by lysosomal acid lipase (LAL) in the lysosome. Free cholesterol moves to the endoplasmic reticulum (ER), re‐esterified into CE by acyl‐coenzyme A:cholesterol acyltransferase (ACAT), and stored in lipid droplets. Cholesterol levels in lipid droplets are regulated by converting CEs back to cholesterol with neutral cholesterol ester hydrolase (nCEH), cholesterol efflux to high‐density lipoproteins (HDLs), and cholesterol transport among lipid droplets, plasma membrane, lysosomes, and mitochondria. Excess cholesterol forms rhomboidal platy crystals on the plasma membrane and 3D irregular crystals on lipid droplets. B) Schematic illustration of the lipid droplet model for cholesterol crystallization visualization. C) The variation in interfacial tension at different cholesterol concentrations of the oil phase ($C_{Chol}^{o}$). The solid lines and shaded regions represent the mean of three trials and the standard deviation, respectively. D,E) Oil/water interfaces without (D) and with cholesterol crystals (E) were examined using bright‐field and BO‐Chol fluorescence imaging. The xz‐plane views represent the 2D cross‐sections at the dotted lines of the xy‐plane images. F) Probability of interfacial cholesterol crystallization at different $C_{Chol}^{o}$ based on multiple experimental trials (N). G) Scheme of growth of a plate‐like cholesterol crystal at the oil/water interface.

However, recent three‐dimensional (3D) Humana Press visualizations of intact cholesterol crystals inside tissues and cells have revealed that intracellular cholesterol crystals grow in morphologies that differ significantly from their known polymorphic shapes.^[^ ^21^, ^22^ ^]^ Unlike typical rhomboid‐shaped cholesterol crystals, intracellular cholesterol crystals exhibit a thin, sheet‐like shape with varying curvatures and rugged edges, forming 3D intertwined agglomerates. In addition, some exhibit a large rod shape and were elongated, extending beyond the size of the cells.^[^ ^8^, ^17^, ^22^ ^]^ Interestingly, these irregularly shaped cholesterol crystals are in contact with lipid droplets, suggesting that unusual crystal morphologies may arise from the unique structural and functional features of the lipid droplets.^[^ ^17^, ^21^ ^]^ Lipid droplets are central organelles that regulate cellular lipid homeostasis through lipid storage and dynamic interactions with other organelles (reviewed in^[^ ^23^ ^]^). They have a spherical hydrophobic core containing neutral lipids such as triacylglycerols and cholesteryl esters, covered by a lipid monolayer primarily composed of phospholipids, free cholesterol, and fatty acids.^[^ ^24^, ^25^ ^]^ In response to cellular cholesterol levels, lipid droplets store free cholesterol on their surfaces and regulate cholesterol concentration through various cellular processes, including intracellular cholesterol trafficking^[^ ^26^, ^27^, ^28^, ^29^, ^30^, ^31^, ^32^ ^]^ and enzymatic reactions (**Figure** **1**A).^[^ ^33^, ^34^, ^35^, ^36^ ^]^ Therefore, an imbalance in cholesterol homeostasis could lead to excessive cholesterol accumulation and crystallization on the lipid droplet surface. However, cholesterol crystallization on the lipid droplet surface has not been explored extensively, and the mechanisms responsible for the distinctive morphologies of intracellular cholesterol crystals remain a mystery.

Here, we reveal how cholesterol crystallizes into non‐rhomboidal crystals on the surface of lipid droplets and eventually evolves into a 3D irregular sheet‐like architecture to understand the non‐traditional morphological growth of cholesterol crystals observed adjacent to lipid droplets. Our findings highlight that the interfacial transport kinetics, unique lipid composition, and curved structure of the lipid droplets significantly impact on interfacial cholesterol crystallization, resulting in the coalescence of non‐rhomboidal platy crystals into 3D sheet‐like agglomerates.

## 2 Results

### 2.1 Nucleation and Growth of Platy Crystals at the Interface

To monitor cholesterol crystallization on the lipid droplet surface in real‐time through confocal fluorescence microscopy with 3D image processing, we established an in vitro reconstitution system that mimics the lipid droplet and its surrounding environment (Figure 1B). This model system comprises three components: nonpolar oil phase, water phase, and oil/water interface. The oil phase mimics the lipid droplet core by containing triolein, cholesterol, cholesteryl esters, and phospholipids. Traces of fluorescently labeled cholesterol (BO‐Chol) and phospholipid (TR‐DHPE) were also included in the oil phase as fluorescence probes for cholesterol and phospholipids. The water phase is a physiologically relevant aqueous buffer solution that contains major salts present in the cytosol at pH 7.4. We formed this model in the 96‐well cell culture plate by simply placing the water phase on the bottom and then gently dropping the oil phase on top of the water phase, which led to the formation of the oil/water interface that mimics the lipid droplet surface.

We first considered the basic system where the oil phase only consisted of triolein and cholesterol. Because of its amphiphilic nature, cholesterol conceivably diffuses from the oil phase to the oil/water interface, forming a lipid monolayer with its hydroxyl group oriented toward the water phase. Based on Fick's first law of diffusion, increasing the cholesterol concentration of the oil phase ($C_{Chol}^{o}$) increases its concentration gradient along the direction of oil‐to‐interface diffusion, potentially leading to an increase in the interfacial cholesterol density (Γ_*Chol*_). Consistent with this expectation, Interfacial tension measurement showed that as $C_{Chol}^{o}$ increased, the interfacial tension at the oil/water interface decreased more rapidly, reaching as low as 10 mN m^−1^ at equilibrium (Figure 1C), which indicates a larger and faster accumulation of cholesterol at the interface with the increase in of $C_{Chol}^{o}$.

To determine if cholesterol that accumulates at the oil/water interface crystallizes, we examined the oil/water interface while increasing $C_{Chol}^{o}$ from 0 mol% to the solubility limit of ≈12 mol% (Figure 1D–F; Figure S1A, Supporting Information). The 2D and 3D BO‐Chol fluorescence images clearly identified the location of the curved oil/water interface due to a significant difference in the BO‐Chol fluorescence intensity between the oil and water phases (Figure 1D). We did not find any crystallization at 7 mol% and below for 14 h (Figure 1F) but observed that at 8 mol%, angular plate‐like crystals began to appear at the oil/water interface with a high probability, forming a clear threshold (Figure 1E). The straight crystal edges were clearly distinguished through bright‐field microscopy because of the different refractive indexes between the crystals and surrounding media. Cholesterol crystals were also detected in fluorescence imaging, with most of them exhibiting a higher BO‐Chol fluorescence intensity than the surrounding media. The cross‐sectional image (xz‐plane) showed that the crystals were located at the oil/water interface and had a platy shape parallel to the interface with a width/thickness ratio of more than 10. This indicates that crystals grow faster parallel to the interface (along the a‐ and b‐axes) than vertically (along the c‐axis) (Figure 1G).

When $C_{Chol}^{o}$ exceeded 8 mol%, the interfacial cholesterol crystals grew larger than the field of view (Figure S1A, Supporting Information). When $C_{Chol}^{o}$ reached 11 mol% or higher, the oil phase became oversaturated with cholesterol (Figure 1F), and needle‐like crystals formed in the oil phase (Figure S1A,B, Supporting Information). These needle‐like crystals are likely to be anhydrous crystalline cholesterol due to the waterless environment of the lipid droplet core (0.1–1 wt.% water/oil content)^[^ ^37^, ^38^ ^]^ and similar morphology to the reported anhydrous cholesterol crystals.^[^ ^39^ ^]^ They nucleate randomly within the volume of the lipid droplet core with random orientation. These results indicate that cholesterol crystallizes first at the surface of the lipid droplet rather than in the core, forming plate‐like crystals oriented parallel to the surface.

### 2.2 Crystal Elongation Depending on Interfacial Cholesterol Diffusion Rate

**Figure 2:**
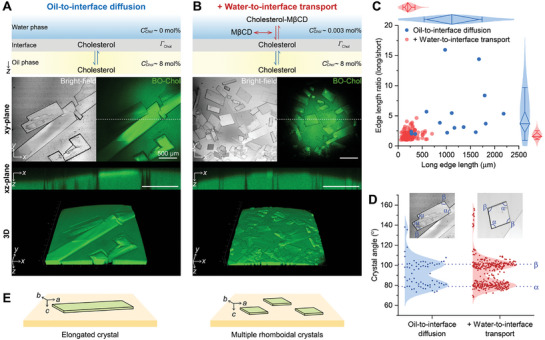
Effect of diffusion kinetics on interfacial cholesterol crystallization. A) Interfacial cholesterol crystals were observed using bright‐field and BO‐Chol fluorescence imaging at $C_{Chol}^{o}$ of 8 mol%. Cholesterol accumulated at the oil/water interface exclusively via only free diffusion from the oil phase to the interface. The xz‐plane views represent the 2D cross‐sections at the dotted lines of the xy‐plane images. B) Cholesterol was additionally transferred to the interface via MβCD‐mediated water‐to‐interface transport. C) The edge length of cholesterol crystals was displayed as a scatter plot. The length ratio of long and short sides was plotted against the long edge length. The distribution of these measurements was approximated using a normal distribution. Results are displayed using diamond box plots, which include a median line and whiskers representing the standard deviation (blue dot, *n* = 15; red dot, *n* = 125). D) The distribution of angles within cholesterol crystals was estimated using the Kernel distribution method. Peaks α and β of the angle distribution were identified at 79° and 101°, respectively, determined through second derivatives of the distribution. The samples analyzed in C and D represent cholesterol crystals that had grown for 12–14 h following the formation of the oil/water interface (blue dot, *n* = 70; red dot, *n* = 375). E) The schematic illustration of elongated and rhomboidal crystals growing along the a‐, b‐, and c‐axes at the oil/water interface. Three axes (a, b, and c) parallel different crystal edges.

To characterize the overall shape of cholesterol crystals, we measured the size and angle of interfacial cholesterol crystals formed at $C_{Chol}^{o}$ of 8 mol% (**Figure** **2**A,C,D). We observed that fewer than ten cholesterol crystals nucleated at the interface, growing gradually larger in different shapes and rates (Figure S2A,B, Supporting Information). The size quantification showed that the cholesterol crystals tended to elongate with various edge ratios between 2 and 20, some of them reaching a few millimeters in edge length (Figure 2C). This elongated morphology differs from the rhomboid‐like shape of triclinic cholesterol monohydrate crystals formed in atherosclerotic lesions^[^ ^8^, ^9^, ^18^ ^]^ and model lipid bilayers.^[^ ^11^, ^13^ ^]^ The elongated crystals may represent either anhydrous crystalline cholesterol or monoclinic cholesterol monohydrate polymorph, as their rod‐like shape is a possible structure of both.^[^ ^8^, ^39^ ^]^ To verify this hypothesis, we examined the X‐ray diffraction (XRD) pattern and angles of the elongated cholesterol crystals. The XRD profile showed the prominent peaks corresponding to the D‐spacing of 34.2 and 17.1 Å (Figure S3, Supporting Information), aligning with the diffraction pattern of cholesterol monohydrate.^[^ ^40^ ^]^ In addition, the elongated crystals exhibited specific bimodal angles of 79° (α) and 101° (β) (Figure 2D), consistent with triclinic cholesterol monohydrate crystals with the largest angle of a unit cell being 100.8°.^[^ ^39^, ^41^ ^]^ Therefore, despite the morphological dissimilarity between the elongated crystals and the typical rhomboidal triclinic cholesterol monohydrate crystals, the elongated crystals are also identified as triclinic cholesterol monohydrate.

Another possibility for morphological change is adsorption‐limited kinetics of crystal growth. Cholesterol crystals can grow at the oil/water interface through a series of steps: cholesterol molecules are adsorbed into the oil/water interface via free diffusion from the oil phase, move to the crystal surface, are stripped from the surrounding solvent molecules, move along the crystal surface to a suitable lattice position, and finally integrate into the crystal structure.^[^ ^42^ ^]^ We hypothesize that if the adsorption of cholesterol molecules to the oil/water interface is slower than their integration into the cholesterol crystals, the concentration gradient along the crystal surface can be generated, potentially leading to the different crystal growth rates along the a‐ and b‐axes and the elongated morphology (Figure 2E). Conversely, when cholesterol adsorption is faster than integration, cholesterol molecules can be evenly distributed on the crystal surface, resulting in comparable growth rates along the a‐ and b‐axes and a rhomboidal crystal shape.

In order to test whether the cholesterol adsorption rate affects the crystal shape, we increased the adsorption rate of cholesterol to the oil/water interface by using MβCD, which encapsulates cholesterol in the water phase and facilitates its reversible transfer it between the water phase and the oil/water interface^[^ ^43^ ^]^ (Figure 2B and S2C). Since MβCD is capable of extracting cholesterol from the cholesterol monolayer and cholesterol crystals into the water phase,^[^ ^13^ ^]^ we saturated MβCD with cholesterol before using it to inhibit the reduction in interfacial cholesterol density and the dissolution of cholesterol crystals. Indeed, during the growth stage of cholesterol crystals, we did not observe any crystal dissolution (e.g., crystal edge roughening and crystal area reduction) with the cholesterol‐saturated MβCD. We confirmed that the cholesterol‐saturated MβCD increased the adsorption rate of cholesterol at the oil/water interface, as indicated by the faster reduction of the interfacial tension to ∼9 mN m^−1^ ≈200 min (Figure S4, Supporting Information). Our imaging showed that the nucleated crystals under the MβCD‐mediated cholesterol transport grew in a rhomboid‐like shape with an edge ratio of ≈2 on average (Figure 2B,C). These rhomboidal crystals were also identified a triclinic cholesterol monohydrate polymorph, as corroborated by the XRD data (Figure S3B, Supporting Information) and the bimodal angle distribution at 79° and 101° (Figure 2D). These findings suggest that crystals adopt a rhomboidal shape under rapid cholesterol adsorption at the oil/water interface, whereas slow adsorption leads to the development of an elongated morphology. This indicates that cholesterol adsorption kinetics at the lipid droplet surface plays a crucial role in regulating crystal morphology and that the fluctuations in interfacial cholesterol density, due to intracellular cholesterol trafficking or enzymatic reactions, may affect the crystal structure and growth dynamics. Moreover, the elongation of cholesterol crystals may underlie the formation of rod‐like structures capable of piercing cell membranes.^[^ ^8^, ^21^, ^22^ ^]^

### 2.3 Variation in Crystal Angle with Cholesteryl Esters

**Figure 3:**
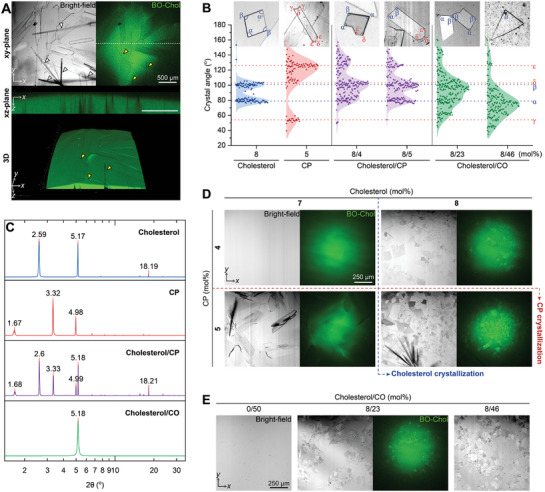
Interfacial cholesterol crystallization with cholesteryl esters. A) Interfacial CP crystallization was observed using bright‐field and BO‐Chol fluorescence imaging. The xz‐plane images represent cross‐sections at the dotted lines of the xy‐plane images. The white and yellow triangles indicate the CP crystals growing non‐parallel to the oil/water interface and their contact sites at the interface, respectively. B) The angle distributions of cholesterol and CP crystals were analyzed using the Kernel distribution. Peaks α and β, corresponding to 79° and 101°, are the angle distribution peaks for cholesterol crystals. Peaks γ, δ, and ε, at 54°, 103°, and 126°, respectively, represent the angle distribution of CP crystals. These peak values were derived from second derivatives of the distribution (from left to right, *n* = 123, 108, 222, 196, 222, 168). The crystals grew for 12–14 h following the formation of the oil/water interface. C) X‐ray diffraction analysis of cholesterol crystals formed in the presence of cholesteryl esters. The statistical analysis by multiple trials is shown in Table S1 (Supporting Information). D,E) Representative bright‐field and fluorescence images of interfacial crystals formed with different concentrations of cholesterol and cholesteryl esters, including CP (D) and CO (E). The oil phase contained cholesterol and cholesteryl esters, while the water phase contained MβCD.

We next addressed the question of whether interfacial cholesterol crystallization is impacted by cholesteryl esters, which are major components of lipid droplets. Cholesteryl esters comprise 10–50% of lipid droplets but are not present in the plasma membranes,^[^ ^44^, ^45^, ^46^ ^]^ potentially explaining the differences in cholesterol crystallization between these locations. We first included cholesteryl palmitate (CP), which is one of the representative saturated cholesteryl esters, in the oil phase. CP mixed well with triolein at CP concentration in the oil phase ($C_{CP}^{o}$) below 5 mol% but formed platy crystals at the oil/water interface at $C_{CP}^{o}$ of 5 mol% or more (**Figure** **3**A; Figure S5, Supporting Information). CP crystals were formed parallel to the interface as well as at angles not parallel to the interface (triangles in Figure 3A). They had non‐uniform shapes with specific three angles of 54° (γ), 103° (δ), or 126° (ε) (Figure 3B) and three main Bragg peaks corresponding to D‐spacings of 52.65, 26.52, and 17.70 Å (Figure 3C; Table S1, Supporting Information), differing from the Bragg peaks of cholesterol crystals.

By controlling both cholesterol and CP concentrations in the oil phase, we confirmed that CP had minimal impact on the critical cholesterol concentration for its interfacial crystallization; cholesterol crystals consistently appeared at $C_{Chol}^{o}$ of 8 mol% or more, independent of CP concentration (Figure 3D). However, in the presence of CP, a significant increase in cholesterol crystal nucleation was observed at the oil/water interface, indicating the potential role of CP as a nucleating agent for cholesterol crystals, which is consistent with the previous computational approach.^[^ ^17^ ^]^ Furthermore, it is notable that cholesterol crystals primarily displayed an irregular, non‐quadrilateral shape, characterized by three dominant angles of ≈ 79°, 101°, and 126° (Figure 3B,D). The difference in the shape of cholesterol crystals with and without CP suggests that cholesterol may co‐crystallize with CP within the same crystal structure. The XRD data corroborate this possibility, showing the Bragg peaks of both triclinic cholesterol monohydrate and CP crystals within the mixed cholesterol crystals formed with CP (Figure 3C; Table S1, Supporting Information). This integrated cholesterol/CP structure is expected to exhibit the physical characteristics of both components, resulting in a combination of angles characteristic of cholesterol crystals (79° and 101°) and CP crystals (103° and 126°).

We also tested another representative cholesteryl ester, cholesteryl oleate (CO). CO is an unsaturated cholesteryl ester and does not crystallize in the oil phase even at a high concentration of 50 mol% at physiological temperature (Figure 3E). Similar to CP, CO promoted cholesterol crystallization, as evidenced by the numerous cholesterol crystals forming at $C_{Chol}^{o}$ of 8 mol%. However, unlike the sharp angle distribution of cholesterol/CP crystals, the cholesterol crystals formed with CO exhibited a broad spectrum of angles (Figure 3B). As the CO concentration increased, the angular distribution shifted lower, centering ≈ 79°, yet remained wide‐ranging. The crystals retained a Bragg peak for cholesterol monohydrate crystals, corresponding to the D‐spacing of 17.1 Å, but the peak at 34.2 Å was no longer observed (Figure 3C; Table S1, Supporting Information). These results suggest that CO disturbs the internal molecular arrangement and symmetry of the crystal structure during its growth. Our experiments demonstrate that cholesteryl esters, including CP and CO, actively influence both the nucleation and the resulting shape of cholesterol crystals, potentially leading to the formation of irregularly shaped triclinic cholesterol monohydrate crystals on the surface of lipid droplets.

### 2.4 Phospholipid‐Mediated Recruitment of Cholesterol to the Interface

**Figure 4:**
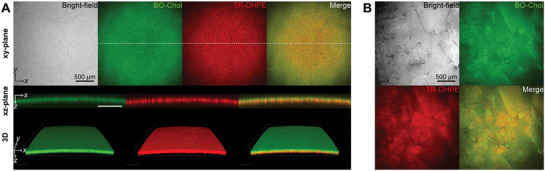
Cholesterol crystallization at the phospholipid‐laden oil/water interface. The oil/water interface was observed using bright‐field and fluorescence imaging in the presence of cholesterol and phospholipids. The concentrations of cholesterol/phospholipids in the oil phase were 8/2 mol% (A) and 10/2 mol% (B). The xz‐plane images represent the cross‐section at the dotted line of the xy‐plane images.

The variation in cholesterol crystallization by cholesteryl esters prompted us to ask whether cholesterol crystallization is also affected by phospholipids that cover the lipid droplet surface. Phospholipids were observed to accumulate at the oil/water interface, as indicated by the high TR‐DHPE fluorescence intensity at the oil/water interface (**Figure** **4**A). BO‐Chol fluorescence images illustrated the colocalization of cholesterol with phospholipids at the interface, suggesting the recruitment of cholesterol to the phospholipid‐laden interface through intermolecular interactions, including hydrogen bonds between the head group of phospholipids and the hydroxyl group of cholesterol.^[^ ^47^ ^]^ The interfacial tension measurement showed that phospholipids and cholesterol rapidly lowered interfacial tension to ≈1 mN m^−1^ (Figure S6, Supporting Information), aligning with the reported surface tension of the lipid droplets (≈1–2 mN m^−1^).^[^ ^48^ ^]^

By controlling the cholesterol concentration, we identified that unlike phospholipid‐free interface, cholesterol did not crystallize at the phospholipid‐laden interface at $C_{Chol}^{o}$ of 8 mol% (Figure 4A) and that a higher $C_{Chol}^{o}$ (10 mol% and more) is required for crystallization (Figure 4B). The inhibitory effect of phospholipids on cholesterol crystal precipitation aligns with the findings of a previous study,^[^ ^49^ ^]^ which demonstrated that phospholipids inhibited the formation of 2D crystalline cholesterol phases in lipid monolayers, as shown by grazing incidence X‐ray diffraction. The cholesterol crystals grew in a quadrilateral shape but had partially rounded angles (Figure 4B; Figure S7, Supporting Information). Initially, the TR‐DHPE fluorescence intensity of the cholesterol crystals was lower than that of the phospholipid‐laden interface, but it gradually increased and eventually surpassed the interface (Figure S7, Supporting Information). This enrichment of phospholipids on the crystal surface represents their preferential interaction with cholesterol crystals, which is consistent with previous observations of phospholipid‐covered triclinic monohydrate crystals.^[^ ^13^ ^]^ Thus, our results suggest that the preferential interaction of phospholipids with cholesterol boosts the interfacial density of cholesterol but inhibits interfacial crystallization and affects the shaping of cholesterol crystals.

### 2.5 Macroscopic Crystal Growing to Sheet Morphology

The cholesterol crystals observed so far are over ten times smaller than our model lipid droplets, but cholesterol crystals can grow to scales larger than lipid droplets in a cell, potentially forming macroscopic structures that could pierce cell membranes.^[^ ^8^, ^21^, ^22^ ^]^ To explore the macroscopic growth process of cholesterol crystals, we developed cholesterol crystals at scales comparable to our model lipid droplets by increasing $C_{Chol}^{o}$ to more than 8 mol% and observed their 3D structure at the oil/water interface. Our visualization showed that interfacial cholesterol crystals can macroscopically develop into flat or curved sheet‐like crystals, depending on the nucleation frequency at the oil/water interface.

**Figure 5:**
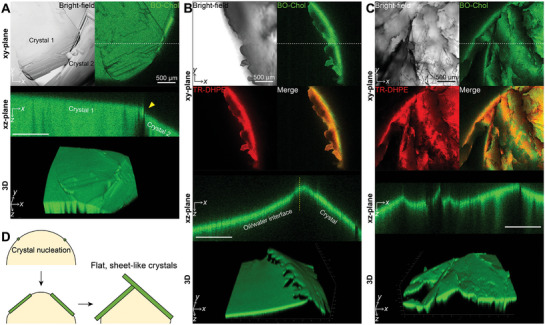
Flat, sheet‐like cholesterol crystals grown at the oil/water interface. A–C) The macroscopically grown cholesterol crystals at the oil/water interface were visualized by the bright‐field and fluorescence imaging at cholesterol/phospholipid concentrations of 9/0 mol% (A), 12/2 mol% (B), and 14/2 mol% (C). The xz‐plane images represent the cross‐section at the dotted line of the xy‐plane image. D) Schematic illustration of the presumable nucleation‐and‐growth process of cholesterol crystals on the lipid droplet surface.

When a few cholesterol crystals occurred at the oil/water interface without MβCD, the crystals grew in a flat platy shape, unaffected by the positive Gaussian curvature of the interface (**Figure** **5**A). The cholesterol crystals flattened the curved oil/water interface upon contact and formed angular interfaces by meeting at non‐parallel angles (yellow triangle in Figure 5A). Although the edge of cholesterol crystals tended to roughen in the presence of phospholipids, cholesterol crystals still expanded 2D into sheet‐shaped forms, altering the adjacent interface curvature from concave to convex along the z‐axis (Figure 5B; Figure S8, Supporting Information). As $C_{Chol}^{o}$ increased, cholesterol crystals expanded to cover most of the interface area (Figure 5C; Figure S9, Supporting Information). When the cholesterol crystals outgrew the limited area of the interface, they were folded and crumpled, resulting in a jagged structure of both cholesterol crystals and interface (Figure 5C). This dynamic deformation of the oil/water interface, observed during the macroscopic sheet‐like crystal growth, is possibly attributed to the extremely low interfacial energy at the phospholipid ‐laden interface (Figure 4B). These results suggest that cholesterol crystals can grow in massive, sheet‐like morphology at the lipid droplet surface, distorting the curvature of the lipid droplets and escaping from the lipid droplets (Figure 5D).

### 2.6 Development of 3D Curved Crystal Agglomerates

**Figure 6:**
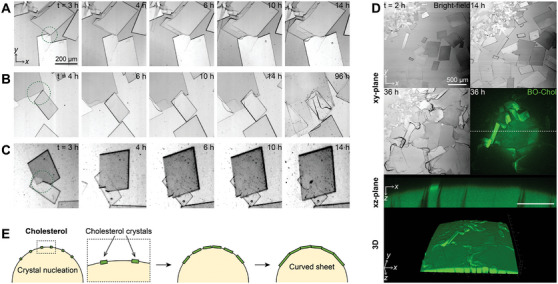
Aggregation of cholesterol crystals into a curved, sheet‐like agglomerate at the oil/water interface. A–C) Cholesterol crystals meet each other at the oil/water interface through lateral merging (A), partial overlap (B), and dissolution (C). D) The aggregation of cholesterol crystals was visualized by the bright‐field and fluorescence imaging at a $C_{Chol}^{o}$ of 9 mol% with the MβCD‐mediated water‐to‐interface transport. E) Schematic illustration of the aggregation of cholesterol crystals on the curved oil/water interface.

However, when many crystals nucleate and grow simultaneously on the confined lipid droplet surface, they are likely to meet and influence the growth process of each other. Real‐time visualization of directly contacting cholesterol crystals revealed that different crystals readily merged upon contact, maintaining their orientation without undergoing 2D rotational movements (**Figure** **6**A). Upon merging, the crystals expanded 2D, filling the interface near the contact point and thereby extending the length of the contact edge. When the crystals partially overlapped, they grew by enlarging the area of overlap (Figure 6B). The crystal overlaps sometimes resulted in the dissolution of one of the crystals (Figure 6C). These results suggest that cholesterol crystals readily combine in a random orientation to form an irregular crystal aggregate, thereby losing original shapes (Figure 6D). As $C_{Chol}^{o}$ increased, the number of nucleated cholesterol crystals increased, leading to their rapid, extensive coalescence at the oil/water interface and the creation of a large, curved, sheet‐like aggregate (Figure S10, Supporting Information). These results suggest that the lateral aggregation of cholesterol crystals on the curved lipid droplet surface promotes the formation of 3D curved sheet‐like crystals (Figure 6E), while an independent cholesterol crystal grows in flat sheet‐like forms.

Considering the extremely low surface energy (γ ∼1 mN m^−1^) and the limited area of the lipid droplet surface, sheet‐like cholesterol crystals may escape from the lipid droplets and merge with those originating from different lipid droplets in the confined intracellular space. This process results in the formation of 3D entangled cholesterol crystal architectures, as observed in previous studies.^[^ ^21^, ^22^ ^]^

## 3 Discussion

This study demonstrates the unique morphological growth processes of cholesterol crystals on the surface of lipid droplets. Our results propose that the lipid droplet surface is an active site in which cholesterol crystallizes into non‐rhomboidal plate‐like crystals and even 3D sheet‐like agglomerates. This provides fundamental insights into understanding the distinctive morphology of intracellular cholesterol crystals observed in recent studies.^[^ ^8^, ^17^, ^21^, ^22^ ^]^ Using an in vitro model lipid droplet system (Figure 1), we highlight that these crystals can grow in different morphologies from the rhomboidal cholesterol crystals originating from the plasma membranes, depending on the unique features of the lipid droplet surface: 1) interfacial cholesterol transport (Figure 2), 2) lipid composition (Figures 3 and 4), and 3) the spherical structure of the lipid droplets (Figures 5 and 6).

Active interactions between lipid droplets and other organelles may also affect cholesterol crystallization. Direct membrane contacts of lipid droplets with the lysosome, endoplasmic reticulum, and mitochondria can cause interfacial lipid transfers on the lipid droplet surface,^[^ ^31^, ^32^ ^]^ potentially inducing the formation or dissolution of cholesterol crystals. Moreover, the hydrolysis of cholesteryl esters to free cholesterol via neutral cholesterol ester hydrolases^[^ ^33^, ^34^, ^35^ ^]^ and lysosomal acid lipase^[^ ^36^, ^50^ ^]^ could influence the cholesterol crystallization by changing the cholesterol accumulation rate and the lipid composition at the lipid droplet surface. Therefore, further studies on the role of other cellular organelles and enzymatic reactions in regulating cholesterol crystallization could provide a more extensive picture of the physiological and pathological contexts.

Cholesterol crystals can nucleate both on the lipid droplet surface (heterogeneous nucleation) and in the core (homogeneous nucleation), but the lipid droplet surface serves as a more active nucleation site for cholesterol crystals than the core (Figure 1F), and these sites produce different structures of cholesterol crystals (Figure S1, Supporting Information). At the lipid droplet surface, free cholesterol accumulating in lipid droplets is recruited to the phospholipid‐laden surface due to its polar hydroxyl group and its preferential interaction with phospholipids (Figure 4). Cholesterol aligns nearly vertically at the oil/water interface, with its hydroxyl group positioned near the phospholipid headgroups. This configuration enables the formation of a cholesterol/phospholipid monolayer. Depending on the lipid composition and surface tension, the cholesterol/phospholipid monolayer can exist as a liquid phase, undergo phase separation into two distinct coexisting liquid phases, or form a crystalline cholesterol phase.^[^ ^16^, ^51^ ^]^ When the cholesterol concentration exceeds a certain threshold (cholesterol/phospholipid ratio > ∼1), cholesterol molecules can spontaneously assemble into a 2D monoclinic cholesterol monohydrate phase in the form of cholesterol multilayers interleaved by an ordered water layer.^[^ ^52^, ^53^ ^]^

Furthermore, cholesteryl esters on the lipid droplet surface facilitate the nucleation of cholesterol monohydrate crystals (Figure 3). Cholesteryl esters are primarily present in the lipid droplet core, but they are partially recruited to the lipid droplet surface with their fatty acid chain oriented toward the water phase,^[^ ^54^, ^55^ ^]^ resulting in the proximity of cholesterol and cholesteryl esters. In particular, saturated cholesteryl esters, such as CP, can spontaneously form a crystalline interdigitated bilayer structure,^[^ ^56^ ^]^ which acts as an epitaxial nucleation site for the monoclinic cholesterol monohydrate phase.^[^ ^17^ ^]^ The monoclinic cholesterol monohydrate domains can either continue to grow into monoclinic cholesterol monohydrate crystals or undergo transformation into triclinic ones,^[^ ^11^, ^53^ ^]^ depending on various environmental factors.^[^ ^8^, ^12^, ^13^, ^17^, ^18^, ^19^, ^20^ ^]^ We found no evidence for the formation of 3D monoclinic structures, including filaments, helices, and tubes,^[^ ^11^, ^12^, ^13^ ^]^ on the lipid droplet surface, suggesting that the lipid droplet surface is the preferred site for the monoclinic‐to‐triclinic phase transformation and the growth of triclinic cholesterol monohydrate crystals.

On the other hand, in the lipid droplet core, higher cholesterol levels are required for cholesterol crystallization, and the core‐derived crystals exhibit a distinctive needle‐like morphology that differs significantly from the surface‐derived platy crystals (Figure S1, Supporting Information). The needle‐like crystals are believed to consist of anhydrous crystalline cholesterol, given the low water content of the lipid droplet core (0.1–1% water/oil content)^[^ ^37^, ^38^ ^]^ and their morphological similarity to previously reported anhydrous cholesterol crystals.^[^ ^39^ ^]^ They nucleate randomly within the volume of the lipid droplet core, with a random orientation. Some of the crystals are oriented nearly perpendicular to the lipid droplet surface and grow in close proximity to the cholesterol monohydrate crystals formed on the lipid droplet surface. This suggests that anhydrous cholesterol and cholesterol monohydrate crystals may form a new type of 3D crystal aggregate; however, the aggregated structure and pathological significance remain unknown.

To the best of our knowledge, it is currently unclear whether different structures of cholesterol crystals have varying consequences for related diseases. Nevertheless, several scenarios can be envisioned as a result of distinct crystal morphologies. First, it has been suggested that the longer the elongated cholesterol crystals are within the cell, the more likely they are to contact and pierce the plasma membranes in the constrained intracellular space.^[^ ^8^, ^21^, ^22^ ^]^ The disruption of the plasma membranes potentially leads to cell death via necrosis, inflammation, and endothelial dysfunction, which could contribute to the development of atherosclerosis. Second, as the cholesterol crystals formed on the lipid droplet surface grow along the surface in a platy or curved sheet‐like shape, they can effectively cover the lipid droplet surface, blocking the trafficking of lipids and metabolites between the lipid droplets and other organelles. This may result in dysregulation of lipid homeostasis and lipid‐induced toxicity.^[^ ^23^ ^]^ Lastly, when cholesterol crystals continue to grow macroscopically and 3D by aggregation in the cytosol, they are more likely to detach from the lipid droplet surface and come into contact with various membrane‐bound organelles, including lysosomes, mitochondria, the endoplasmic reticulum, and the Golgi apparatus. Given that cholesterol crystals extract cholesterol from the plasma membrane upon contact,^[^ ^57^ ^]^ it is plausible that the binding of cholesterol crystals to such organelles may precipitate sudden membrane destabilization and catastrophic organelle malfunction. Therefore, different morphologies of intracellular cholesterol crystals may lead to different outcomes, but further studies are required to determine the correlation between crystal morphology and pathological consequences.

In summary, this study elucidates the 3D morphological growth dynamics of cholesterol crystals nucleating from the surface of lipid droplets. Real‐time imaging with a meticulously controlled lipid droplet model system has uncovered significant influences of interfacial transport, lipid composition, and the geometry of the lipid droplet surface on the crystallization and shaping of cholesterol crystals. Our results provide novel insights into the fundamental mechanisms of the formation and non‐traditional growth of cholesterol crystals, distinct from those derived from plasma membranes.

## 4 Experimental Section

### Materials

Triolein, cholesterol, cholesteryl palmitate, and cholesteryl oleate were purchased from Sigma‐Aldrich. Phospholipids including 1,2‐dioleoyl‐sn‐glycero‐3‐phosphocholine (DOPC), 1,2‐dioleoyl‐sn‐glycero‐3‐phosphoethanolamine (DOPE), and 23‐(dipyrrometheneboron difluoride)‐24‐norcholesterol (BO‐Chol) were purchased from Avanti Polar Lipids. The fluorescent phospholipid probe, Texas Red 1,2‐dihexadecanoyl‐sn‐glycero‐3‐phosphoethanolamine (TR‐DHPE), was purchased from Thermo Fisher Scientific. Components for the aqueous phase, including sodium chloride, potassium chloride, magnesium chloride, 4‐(2‐Hydroxyethyl)piperazine‐1‐ethanesulfonic acid (HEPES), and methyl‐beta‐cyclodextrin (MβCD), were purchased from Sigma–Aldrich. Experiments were conducted in transparent 96‐well cell culture plates purchased from SPL Life Sciences.

### Preparation of Lipid Droplet Model System and Visualization

The oil phase mimics the lipid droplet core, consisting of triolein (46‐100 mol%), cholesteryl esters (0–46 mol%), cholesterol (0–14 mol%), and phospholipids (0–2 mol%), where phospholipids consisted of 1.5 mol% DOPC and 0.5 mol% DOPE. To monitor the distributions of cholesterol and phospholipids, BO‐Chol and TR‐DHPE were added to the oil phase at a concentration of 2 × 10^−4^ and 5 × 10^−3^ mol%, respectively. BO‐Chol had no significant impact on the crystal morphology and nucleation‐to‐growth dynamics (Figure S11, Supporting Information). The lipid‐containing oil mixture was incubated at 50 °C for 1 h to ensure complete dissolution of the lipids, and then cooled down to 37 °C. The cytosol‐mimicking aqueous phase included 12 mm sodium chloride, 145 mm potassium chloride, 2 mm magnesium chloride, and 10 mm HEPES, adjusted to pH 7.4.

In order to induce interfacial cholesterol transport between the water phase and interface, cholesterol and MβCD were added to the water phase at concentrations of 0.15 and 3 mm (1:20 mol%), respectively. At this concentration condition, cholesterol was almost saturated in the water phase. The MβCD‐mediated cholesterol transport barely changed the cholesterol concentration in the bulk oil phase (8 mol%) because the amount of cholesterol encapsulated by MβCD in the water phase is extremely low (≈0.003 mol%).

For the experimental setup and imaging, the transparent 96‐well cell culture plate was placed on the stage of confocal microscope (STELLARIS 5, Leica) and heated to 37 °C with the Stage‐top Incubator System T (Live Cell Instrument). Initially, 100 µl of the aqueous phase was injected on bottom of a well of the cell culture plate and allowed to equilibrate to 37 °C. Then, 400 µl of the oil phase was gently deposited on the water phase to establish the oil/water interface, marking the onset of the experiment (t = 0). Cholesterol crystals formed at the oil/water interface were typically visualized for 14 h through combined bright‐field and fluorescence imaging modes. 3D images were acquired by a z‐stack scan mode. The crystal sizes and angles in the obtained images were analyzed by ImageJ software.^[^ ^58^ ^]^

### Pendant Drop Analysis

Interfacial tension measurements were performed using the pendant drop method. Using the SEO Phoenix 300 Touch equipment, droplets from the water phase were suspended in the lipid oil phase composition described previously. Images of the droplet were captured at intervals of several seconds using the equipment, continuing until the droplet shape stabilized. The recorded images were then analyzed to determine the interfacial tension. Calculations were conducted using Surfaceware 9 software, supplemented with custom scripts written in MATLAB. To ensure the reproducibility of the results, the entire sample preparation and measurement procedures were repeated at least three times.

### X‐Ray Diffraction (XRD)

XRD analysis was performed using a Rigaku Smartlab High‐resolution Powder X‐ray diffractometer with a Cu Kα radiation source (λ = 1.5406 Å). Theta‐theta measurements spanned a 2θ range from 1° to 80°, employing a step size of 0.01° and a scan rate of 10° per minute. The diffractometer was operated at 45 kV and 200 mA, under room temperature conditions. For the sample preparation, crystals formed at the oil/water interface were carefully transferred onto filter paper and isolated by removing the water and oil using a vacuum freeze dryer. The remaining crystal samples on the filter paper were then mounted on zero‐background silicon sample holders designed for XRD analysis. Following data collection, XRD patterns were analyzed using Rigaku XRD PDXL software. Both first and second derivative analyses of the peaks were also checked to find hidden peaks. To ensure the reproducibility of the results, the entire sample preparation and measurement procedures were repeated at least three times.

### Statistical Analysis

The data presented as means ± standard deviations were analyzed from at least three independent experiments to ensure robustness. The number of trials and sample size (*n*) for each analysis are provided in the figure legends, the Experimental section, and the Supporting Information. Crystal angle distributions and peak values were analyzed using OriginLab software.

## Conflict of Interest

The authors declare no conflict of interest.

## Author Contributions

H.R.L. and S.K. contributed equally to this work. H.R.L., S.K., and S.Q.C. conceived the idea and designed the research. H.R.L. and S.K. performed the experiments and analyzed data. H.R.L., S.K., and S.Q.C. wrote the manuscript.

## Supporting information



Supporting Information

## Data Availability

The data that support the findings of this study are available from the corresponding author upon reasonable request.
