# Supplementary material for: Lipid Droplet Surface Promotes 3D Morphological Evolution of Non‐Rhomboidal Cholesterol Crystals
Source: Adv Sci (Weinh). 2024 Nov 8;12(1):2409201. doi: 10.1002/advs.202409201 (PMC11714234; doi:10.1002/advs.202409201)
Supplement: Supplementary file 1 — Supporting Information [file ADVS-12-2409201-s001.pdf]

ADVANCED SCIENCE
Open Access

Supporting Information

for *Adv. Sci.*, DOI 10.1002/advs.202409201

Lipid Droplet Surface Promotes 3D Morphological Evolution of Non-Rhomboidal Cholesterol Crystals

*Hyun-Ro Lee, Seunghan Kang and Siyoung Q. Choi**

Supporting Information

Title

Lipid Droplet Surface Promotes Three-dimensional Morphological Evolution of Non-rhomboidal Cholesterol Crystals

*Hyun-Ro Lee, Seunghan Kang, Siyoung Q. Choi**

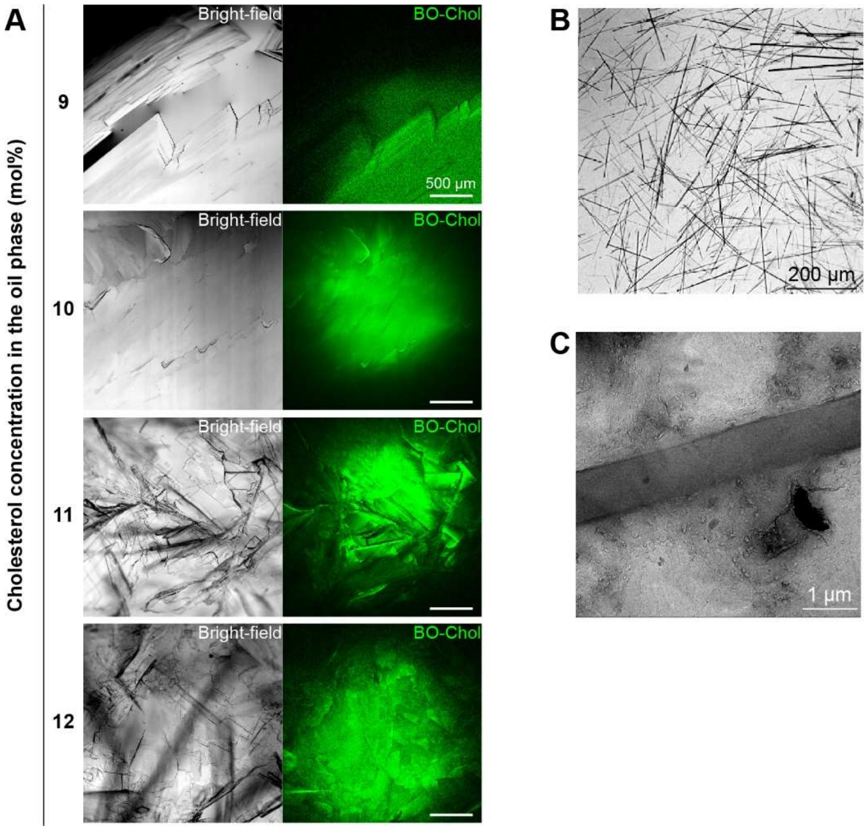

Figure S1. Cholesterol crystallization at the oil/water interface and in the oil phase. (A) Representative images of cholesterol crystals formed at the oil/water interface at the cholesterol concentration in the oil phase of between 9-12 mol%. (B-C) Needle-like cholesterol crystals formed in the oil phase were extracted from the oil phase and visualized through the bright-field microscopy (B) and cryo-transmission electron microscopy (Cryo-TEM) (C). Cryo-TEM measurements were performed using a Thermo Scientific Glacios Cryo-TEM equipped with a HAADF STEM detector. The microscope was operated at an acceleration voltage of 200 kV, and the samples were maintained at -170°C throughout the measurement. The images were recorded with a defocus value range from -0.5 µm to 1.0 µm.

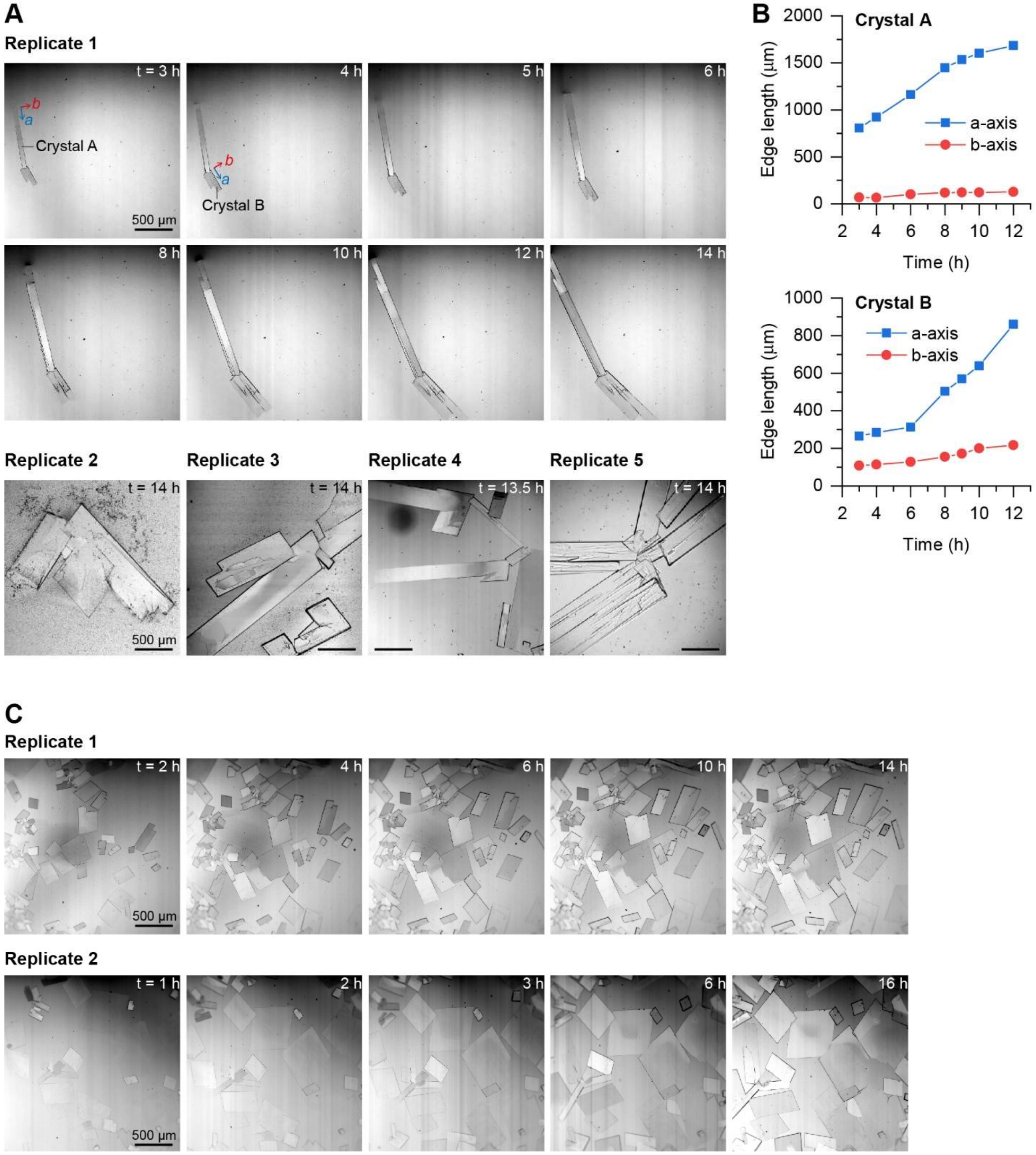

Figure S2. Representative images of interfacial cholesterol crystals. (A) Crystals formed by oil-to-interface adsorption at a cholesterol concentration in the oil phase (C_{Chol}^{o}) of 8 mol%. The replicates 2 and 3 are the same crystals shown in Figure 1E and 2A, respectively. (B) The edge lengths of cholesterol crystals (Replicate 1) along the a- and b-axes were measured over time. The crystal growth rate along the a-axis is significantly greater than that along the b-axis, leading to the elongated crystal morphology. (C) Crystals formed by MβCD-mediated cholesterol transport at C_{Chol}^{o} of 8 mol%. The replicate 1 is the same crystals shown in Figure 2B. t represents the time measured from when the oil/water interface was formed.

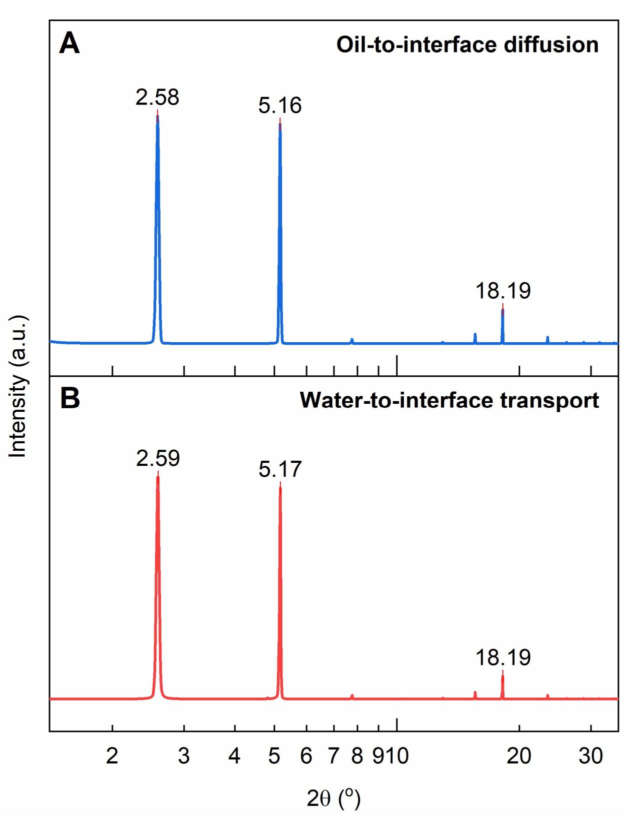

Figure S3. X-ray diffraction patterns of cholesterol crystals. Crystals formed by oil-to-interface diffusion (A) and MβCD-mediated water-to-interface transport (B).

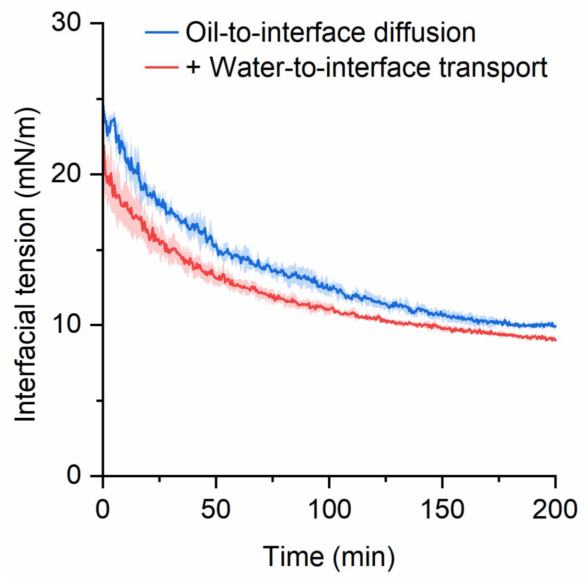

Figure S4. Variation in interfacial tension by adsorption of cholesterol at the oil/water interface. Interfacial tension was quantified using a pendant drop tensiometer, varying according to the different interfacial processes described in the main text. C_{Chol}^{o} was set at 4.5 mol%. The shaded regions represent the standard deviation.

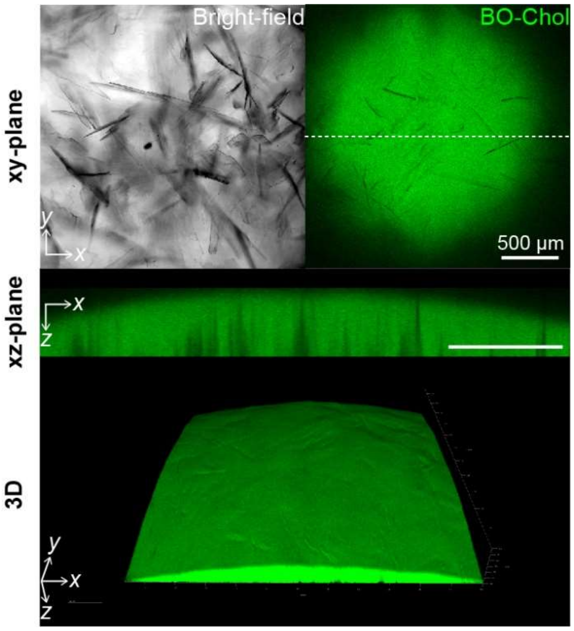

Figure S5. Crystallization of cholesteryl palmitate (CP) at the CP concentration in the oil phase of 6 mol%. CP crystals were visualized through bright-field and BO-Chol fluorescence imaging at the oil/water interface. The xz-plane image corresponds to the cross section at the dotted line of the xy-plane image.

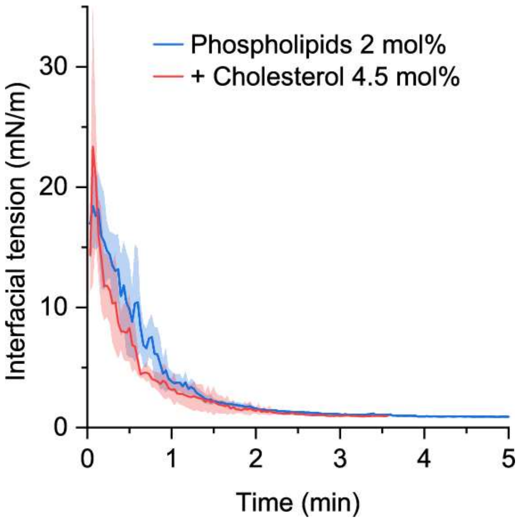

Figure S6. Variation in interfacial tension by adsorption of phospholipids and cholesterol at the oil/water interface. Interfacial tension at the phospholipid-laden interface was assessed using a pendant drop tensiometer. The shaded regions represent the standard deviation.

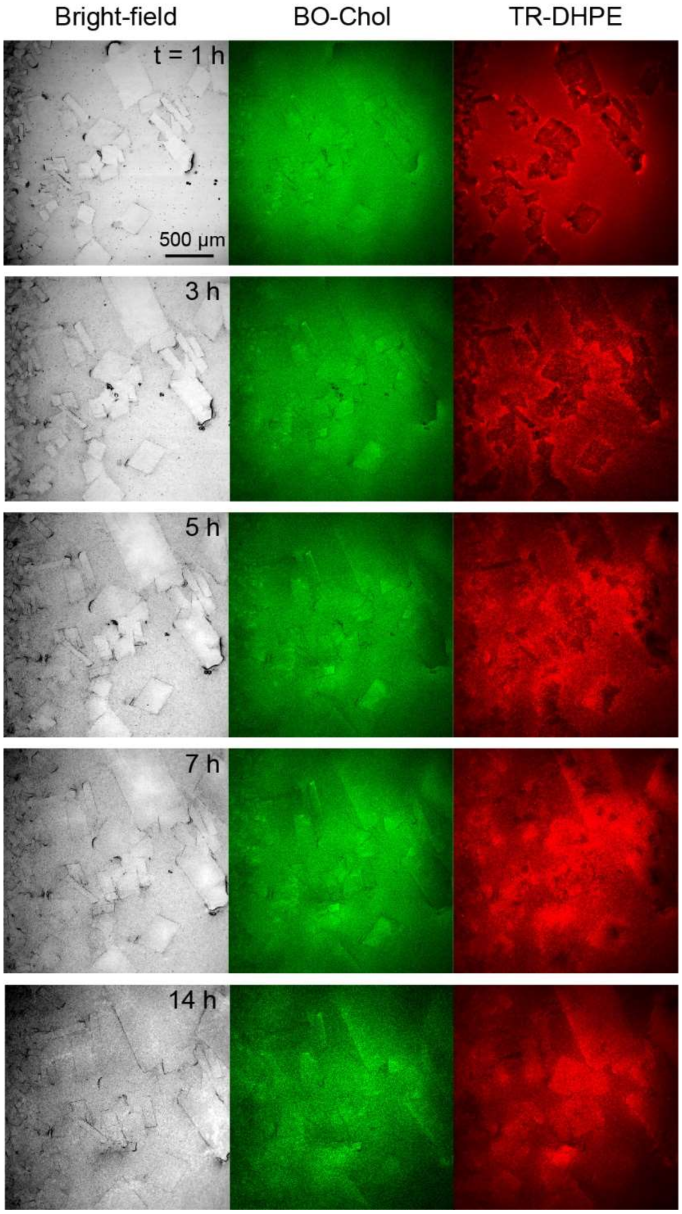

Figure S7. Temporal growth of cholesterol crystals at the phospholipid-laden oil/water interface. The crystals are identical to those shown in Figure 4C. t represents the time measured from when the oil/water interface was formed.

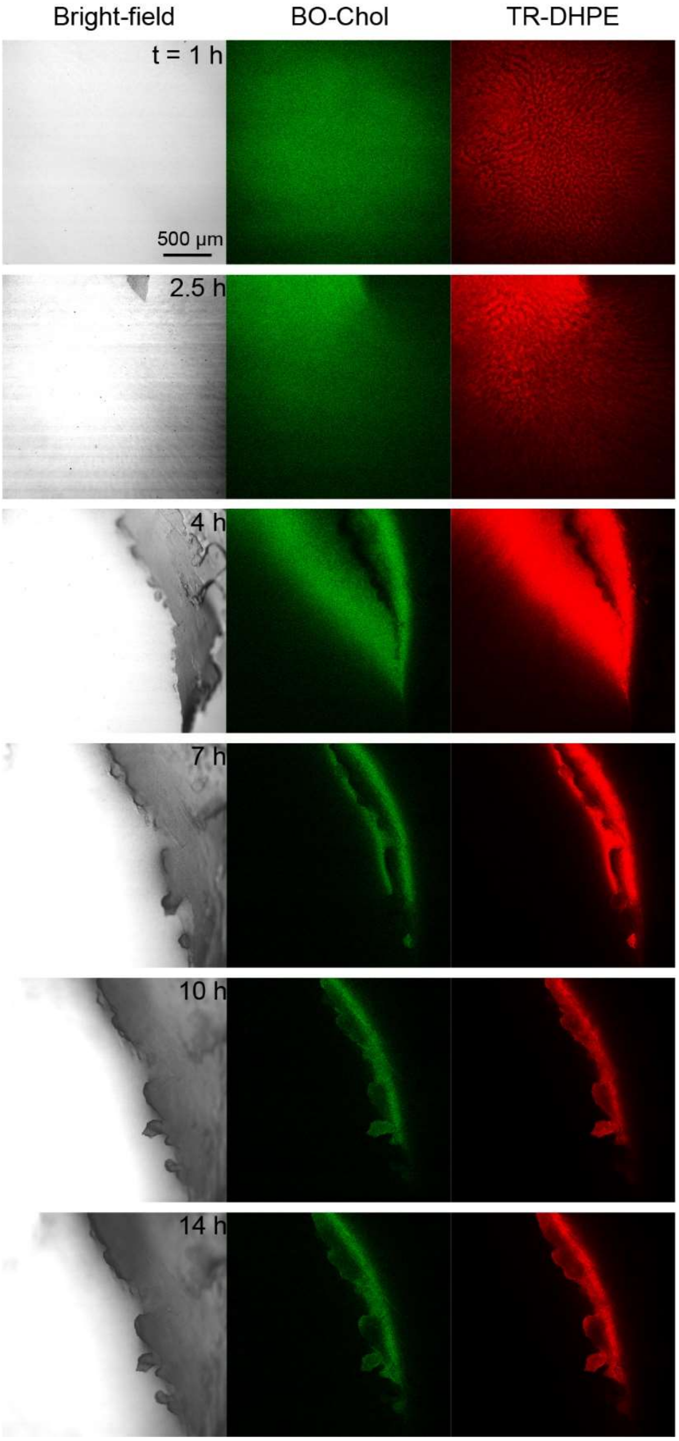

Figure S8. Temporal growth of flat, sheet-like cholesterol crystals at the phospholipid-laden oil/water interface. The crystals are identical to those shown in Figure 5C. t represents the time measured from when the oil/water interface was formed.

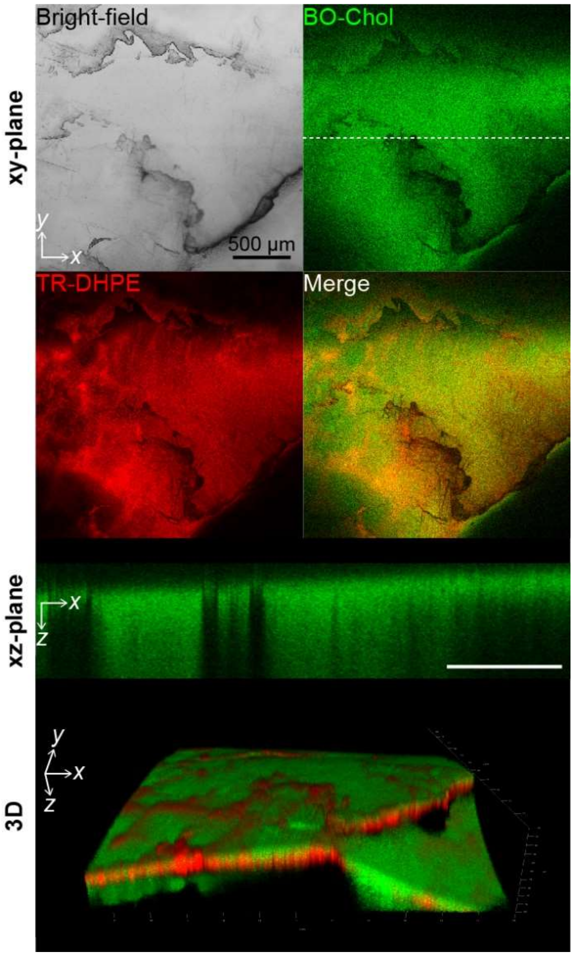

Figure S9. Flat, sheet-like cholesterol crystals grown at the phospholipid-laden oil/water interface. The interfacial cholesterol crystals were visualized by the bright-field and fluorescence imaging at cholesterol/phospholipid concentrations of the oil phase of 13/2 mol%. The xz-plane view represents the cross-section at the dotted line of the xy-plane image.

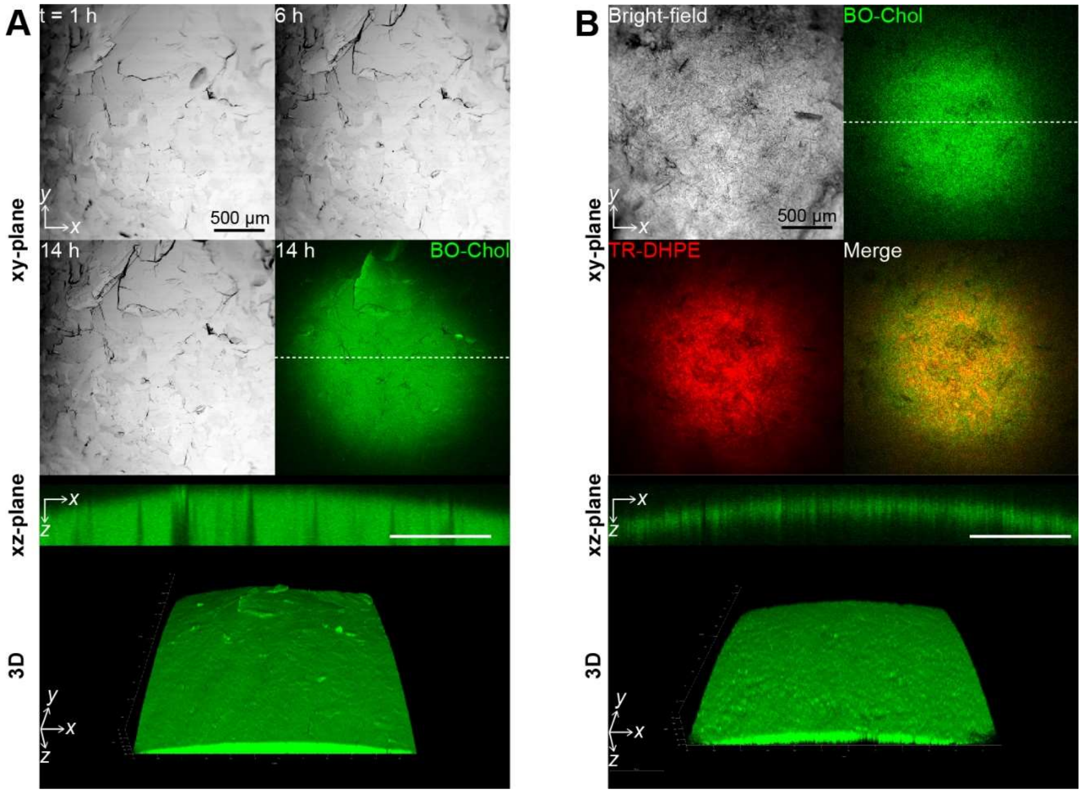

Figure S10. The formation of curved, sheet-like cholesterol crystal aggregates at the oil/water interface. Crystals were visualized through bright-field and BO-Chol fluorescence imaging at cholesterol/phospholipid concentrations of 10/0 mol% (A) and 12/2 mol% (B) in the presence of MβCD. The xz-plane image represents to the cross section at the dotted line of the xy-plane image. t represents the time measured from when the oil/water interface was formed.

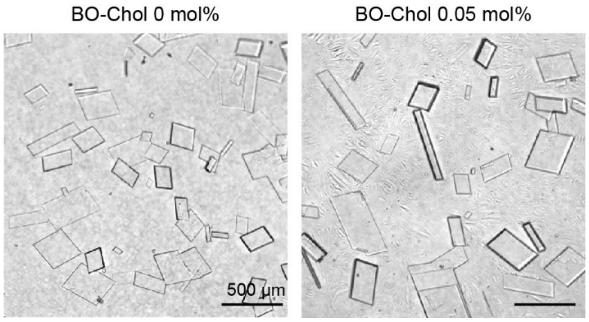

Figure S11. The effect of BO-Chol on interfacial cholesterol crystallization. To determine whether the addition of BO-Chol influences cholesterol crystallization, a controlled experiment was conducted in which cholesterol crystallization was visualized with 0 mol% and 0.05 mol% of BO-Chol. The images were observed by bright-field imaging. The crystallization dynamics and morphology of cholesterol crystals were consistent between systems with and without BO-Chol, indicating that trace amounts of BO-Chol have a negligible effect on crystal morphology.

Table S1. Bragg peaks and D-spacings of cholesterol crystals formed with cholesteryl esters

Crystals	2θ [°][a)]	D-spacing [Å] [a)]	# of trials
**Cholesterol**	2.59 ± 0.02	34.20 ± 0.11	3
	5.17 ± 0.02	17.09 ± 0.02	3
	18.19 ± 0.02	4.87 ± 0.00	3
**CP**	1.68 ± 0.00	52.65 ± 0.15	5
	3.33 ± 0.01	26.52 ± 0.06	5
	4.99 ± 0.01	17.70 ± 0.03	5
**Cholesterol/CP**	1.67 ± 0.00	52.76 ± 0.14	4
	2.60 ± 0.01	34.01 ± 0.11	4
	3.33 ± 0.00	26.52 ± 0.03	4
	4.99 ± 0.00	17.71 ± 0.02	4
	5.18 ± 0.01	17.05 ± 0.04	4
	18.20 ± 0.01	4.87 ± 0.00	4
**Cholesterol/CO**	5.17 ± 0.01	17.07 ± 0.03	3

[a)]Expressed as mean ± standard deviation
